# Selected adjuvants increase the efficacy of foliar biofortification of iodine in bread wheat (*Triticum aestivum* L.) grain

**DOI:** 10.3389/fpls.2023.1246945

**Published:** 2023-09-20

**Authors:** Esther Magor, Matthew Deas Wilson, Henri Wong, Tom Cresswell, José Tonatiuh Sánchez-Palacios, Richard William Bell, Beth Penrose

**Affiliations:** ^1^ Tasmanian Institute of Agriculture, University of Tasmania, Hobart, TAS, Australia; ^2^ Australian Nuclear Science and Technology Organisation, Sydney, NSW, Australia; ^3^ Centre for Sustainable Farming Systems, Food Futures Institute, Murdoch University, Murdoch, WA, Australia; ^4^ SoilsWest, Murdoch University, Murdoch, WA, Australia

**Keywords:** biofortification, nutrient deficiency, iodine, wheat grain, adjuvants, organosilicon surfactants

## Abstract

Agronomic biofortification of crops is a promising approach that can improve the nutritional value of staple foods by alleviating dietary micronutrient deficiencies. Iodine deficiency is prevalent in many countries, including Australia, but it is not clear what foliar application strategies will be effective for iodine fortification of grain. This study hypothesised that combining adjuvants with iodine in foliar sprays would improve iodine penetration in wheat, leading to more efficient biofortification of grains. The glasshouse experiment included a total of nine treatments, including three reference controls: 1) Water; 2) potassium iodate (KIO_3_) and 3) potassium chloride (KCl); and a series of six different non-ionic surfactant or oil-based adjuvants: 4) KIO_3_ + BS1000; 5) KIO_3_ + Pulse^®^ Penetrant; 6) KIO_3_ + Uptake^®^; 7) KIO_3_ + Hot-Up^®^; 8) KIO_3_ + Hasten^®^ and 9) KIO_3_ + Synerterol^®^ Horti Oil. Wheat was treated at heading, and again during the early milk growth stage. Adding the organosilicon-based adjuvant (Pulse^®^) to the spray formulation resulted in a significant increase in grain loading of iodine to 1269 µg/kg compared to the non-adjuvant KIO_3_ control at 231µg/kg, and the water and KCl controls (both 51µg/kg). The second most effective adjuvant was Synerterol^®^ Horti Oil, which increased grain iodine significantly to 450µg/kg. The Uptake^®^, BS1000, Hasten^®^, and Hot-Up^®^ adjuvants did not affect grain iodine concentrations relative to the KIO_3_ control. Importantly, iodine application and the subsequent increase in grain iodine had no significant effects on biomass production and grain yield relative to the controls. These results indicate that adjuvants can play an important role in agronomic biofortification practices, and organosilicon-based products have a great potential to enhance foliar penetration resulting in a higher translocation rate of foliar-applied iodine to grains, which is required to increase the iodine density of staple grains effectively.

## 1 Introduction

Iodine is essential for human nutrition. Although it is reported to be beneficial to plants (Kiferle et al., 2021), it is not considered an essential element for plant growth and health. As it is a trace element in the environment, most plant-based foods, including cereal grains, contain very low concentrations of iodine (Fordyce, 2003; Haldimann et al., 2005). Much of the iodine in soils is not from the soil parent material, but rather from rainfall from the ocean (Fuge and Johnson, 2015). Thus, distance to the coast (Saiz-Lopez and Plane, 2004), rainfall patterns, and the soil’s ability to retain iodine (Bowley, 2013) are important factors influencing the distribution and amount of iodine in terrestrial environments that can be taken up by crops. Multiple studies (Medrano-Macías et al., 2016; Cakmak et al., 2017; Gonzali et al., 2017) suggest that biofortification of widely consumed staple foods such as wheat is a promising approach for reducing the burden of iodine deficiency for humans. Potential disadvantages of biofortification are the requirements for frequent applications of costly fertilisers, which could decrease crop profitability and supply excess nutrient into the environment (Samoraj et al., 2018). Also, food processing may decrease the bioavailability of the increased micronutrient levels present in biofortified crops, and challenges have been identified in ensuring effective coverage, accessibility and equity within large-scale fortification programs (Osendarp et al., 2018). Therefore, it is important to determine what practical approaches to biofortification will optimise wheat grain iodine concentrations so it can be implemented in food crop systems as a strategy to mitigate the risks of insufficient dietary intake of iodine.

Due to low soil supply of plant available iodine and the interference of soil constraints on root uptake, foliar uptake is considered as an important pathway of plant uptake of iodine from the environment (Whitehead, 1984; Fuge and Johnson, 2015). Iodine enters leaf tissues by penetrating leaf cuticles or diffusing through stomata (Fernández and Eichert, 2009). Foliar uptake can be directly from the atmosphere or from iodine-containing gases and particulate matter deposited on plant surfaces. Iodine deposition on leaf surfaces depends on leaf morphology and stem architecture. For example, Heinemann and Vogt (1980) found that leaf angle and leaf area were responsible for clover absorbing twice as much iodine as grass. Once within leaf tissues, iodine is likely transported to vascular tissues for re-distribution via similar processes that occur in roots involving chloride channels permeable to iodine anions (Skerrett and Tyerman, 1994; Roberts, 2006; White and Broadley, 2009) or ATP-dependent proton pumps (Gonzali et al., 2017). However, this transport may be hindered by the organophilic nature of iodine anions that bind to the cuticular waxes of leaf surfaces (Shaw et al., 2007).

Adjuvants are additives added to agrochemical spray mixes with the purpose of enhancing the foliar use efficiency of nutrients and other agrichemicals applied to plants, primarily by lowering surface tension. They help improve the economic viability and environmental sustainability of agrochemical applications by reducing the amount of active ingredient required (Castro et al., 2014). Most common adjuvants used in agriculture are non-ionic surfactants (Somervaille et al., 2011); however ionic and various oil-based products are also used. In this study, non-ionic surfactants (including an organosilicon) and oil-based adjuvants were trialled for their efficacy in improving iodine uptake in foliar sprays. Organosilicons are a novel class of non-ionic surfactant which have been termed ‘super surfactants’ (Chen et al., 2018) and have been shown to significantly lower surface tension (Knoche, 1994; Schönherr et al., 2005) and possess extreme leaf-wetting, spreading and penetrating capability (Stevens, 1993; Knoche, 1994). In line with the standard adjuvant terminology outlined by the American System for Testing Materials, the Australian Pesticides and Veterinary Medicines Authority classifies organosilicons as both wetters/spreaders and penetrants, while non-ionic surfactants are classified as wetters/spreaders, and oils are classified as penetrants (APVMA, 2022; ASTM, 2022). Oil-based adjuvants are usually derived from crude oils such as petroleum products or from biological oil concentrates. They improve penetration through leaf cuticles, reduce evaporation once droplets leave sprayers, and reduce drying speed of droplets on surfaces to extend the active life of agrochemicals (Pacanoski, 2015; Congreve and Cameron, 2019). Many commercial products are a combination of surfactants and oils, but there is little information about the role or efficacy of adjuvants in fortification of grains with iodine. Identifying appropriate adjuvants for use with KIO_3_ applied to wheat could improve the practical feasibility, economic viability, and environmental sustainability of agronomic biofortification as a strategy to mitigate against dietary iodine deficiency.

This project aimed to explore the potential of spray adjuvants for optimising foliar biofortification of a popular high-yielding Australian wheat variety with iodine. We hypothesised that adjuvants would facilitate the loading of iodine in wheat grain.

## 2 Materials and methods

### 2.1 Experimental site

All plants were grown under glasshouse conditions at the Tasmanian Institute of Agriculture’s Horticultural Research Centre at the University of Tasmania’s Sandy Bay campus (Tasmania, Australia). Over the course of the trial, the minimum glasshouse temperature recorded was 4.4°C and the maximum 29.4°C.

### 2.2 Experimental set-up and management

Four seeds of a widely-cultivated, fast-maturing and high yielding (Zaicou-Kunesch et al., 2017; Wheeler, 2018) cultivar of winter wheat in Australia (*Triticum aestivum* L. cv. Mace) were sown into 4L plastic pots filled with a 70:30 nutrient poor sand-perlite mix on the 16^th^ of March 2022. Once established, seedlings were thinned to two plants per pot. Before sowing, seeds were sterilised by soaking in sodium hypochlorite (4% v/v in water) solution for ten minutes and rinsing in deionised water multiple times.

Until seedlings emerged, pots were hand-watered daily. From two weeks after sowing, water and nutrients (Hoagland solution) (Hoagland and Arnon, 1950) were supplied to pots via drip fertigation at a daily rate of approximately 30 mL per pot, delivered for a duration of two minutes, five times per day (at 6:00, 9:00, 12:00, 15:00, and 18:00). The total daily application rate was reduced when the duration of the 15:00 and 18:00 injection periods was shortened to one minute at ten weeks after sowing. The fertigation tank contained Hoagland solution at half-strength during seedling growth stages and full strength from four weeks after sowing. Throughout the experiment, pots were periodically flushed with tap water to prevent nutrient build up. The fertigation system was shut off on the 26^th^ of July 2022 to arrest vegetative growth and promote even grain ripening and crop desiccation.

### 2.3 Experimental design

The experiment utilised a single-factor experimental design with nine foliar spray treatments (**Table 1**) replicated six times, creating a total of fifty-four independent pots. All products are commonly used in industry to improve the efficiency of applying foliar agrochemical sprays to broadacre crops. All pots were arranged on raised benches in a randomized complete block design with each bench representing one block.

**Table 1:** Details of control and iodine fortification treatments applied; including classification, active ingredients, and application rate of adjuvants.

Treatment	Adjuvant	Chemical class	Active ingredient/s (AI)	Application rate:		
				Label directions	mL/L	AI g/L
Water	N/A					
Potassium iodate (KIO_3_)	N/A					
Potassium chloride (KCl)	N/A					
KIO_3_ + BS1000	BS1000	Alcohol alkoxylate non-ionic surfactant	1000 g/L alcohol alkoxylate	2mL/15L	0.135	0.135
KIO_3_ + Pulse^®^ Penetrant	Pulse^®^	Organosilicone non-ionic surfactant	1000g/L modified polydimethylesiloxane	200mL/100L	2	2
KIO_3_ + Uptake^®^	Uptake^®^	Non-ionic surfactant and paraffinic (petroleum-based) oil	582g/L paraffinic oil 240 g/L alkoxylated alcohol non-ionic surfactants	500mL/100L	5	2.91 1.2
KIO_3_ + Hot-Up^®^	Hot-Up^®^	Non-ionic surfactant and mineral (petroleum-based) oil	340 g/L non-ionic surfactant blend 190 g/L mineral oil 140 g/L ammonium sulphate	0.25-1.0L/100L	6.25	2.125 1.19 0.875
KIO_3_ + Hasten^®^	Hasten^®^	Esterified vegetable (plant-based) oil	704 g/L ethyl and methyl esters of canola oil fatty acids 196 g/L non-ionic surfactants	200-500mL/100L	3.5	2.46 0.69
KIO_3_ + Synerterol^®^ Horti Oil	Synerterol^®^ Horti Oil	Botanical (plant-based) oil concentrate	850g/L emulsifiable botanical oil	30-50mL/10L	4	3.4

All information sourced from product labels (Corteva Agriscience, 2023; Nufarm, 2023a; Nufarm, 2023b; Organic Crop Protectants, 2023; Vicchem, 2023a; Vicchem, 2023b)

N/A, not applicable.

### 2.4 Treatment preparation and application

All KIO_3_ was applied at a rate of 0.05% w/v or 0.5 g/L, equivalent to 2.3 mM iodine, following the recommendations of Cakmak et al. (2017). The KCl treatment acted as a reference control to account for a confounding effect of potassium in wheat plants treated with KIO_3._ KCl was applied at 0.174 g/L to achieve the same concentration of potassium in solution. Adjuvants were applied with KIO_3_ at rates according to directions recommended by manufacturers (**Table 1**). All spray solutions were prepared in separate 2 L handheld pressure sprayers.

Handheld pressure sprayers were used to apply foliar spray solutions to all above-ground plant parts until complete coverage of leaf surfaces was attained. To prevent cross contamination between treatments due to spray drift, treatment groups were sprayed one at a time. For each treatment, all six replicates were brought outside the glasshouse and sprayed together. Once treated, all pots of the same treatment group were moved to a separate area in the glasshouse and left to dry for at least one hour. Pots were then returned to their allocated benches but randomly re-positioned within benches. The first spray application occurred on the 9^th^ of May (approximately eight weeks after sowing) when the majority of plants had reached the heading growth stage - Zadoks growth scale 50 [ZGS 50 (Zadoks et al., 1974)]. The second application occurred on the 28^th^ of June (approximately fifteen weeks after sowing) when most plants were at early milk development (ZGS 73). The weather conditions were suitable for spraying on both dates, with no rainfall, strong winds, or extreme heat.

### 2.5 Measurements of plant health

We estimated foliar chlorophyll content in wheat leaves as an indicator of plant health. A handheld SPAD-502 Plus Chlorophyll Meter (Konica Minolta, Japan) was used to measure the relative concentration of chlorophyll per leaf area. SPAD index values were obtained for three randomly sampled leaves from each plant. The recorded SPAD index value was an average of data collected from three different points along the length of sampled leaves. SPAD values were measured just prior to each of the treatment applications, approximately one week after each treatment application, and about half-way between the two treatment applications (8, 9, 12, 15, and 16 weeks after sowing).

### 2.6 Harvest and measurements of plant productivity

All plants were harvested on the 24th of August, 23 weeks after the sowing date. Using secateurs, plants were cut at ground level, and seed heads cut off just below the basal spikelets. Seed heads and vegetative tissues were placed in separate, clearly labelled paper bags. The number of tillers and heads per pot were recorded.

To prevent any potential loss of iodine via volatilization, all samples were left to air dry in a glasshouse for one week (approximate temperature range of 8-25°C). Fans were set up to maintain airflow and plastic sheets were placed over the samples to keep off any moisture from the overhead irrigation in other parts of the glasshouse. Dried seed heads were threshed using a Kimseed Multi-Thresher (Kimseed Australia Pty Ltd, Australia) and 5 mm sieves and a 757 South Dakota Seed Blower were used to remove chaff material from the seed.

The total grain yield (g) per pot was measured and the number of seeds was counted using a seed counter. Harvest index was calculated as the ratio of grain produced to total shoot dry weight.

### 2.7 Iodine extraction method validation using certified reference material

Harvested grain samples were freeze-dried and milled with a ball mill.

Triple quadrupole inductively coupled plasma – mass spectrometry (TqICP-MS; Agilent 8900, Agilent Technologies, USA) was used to determine the concentration of iodine extracted from samples with tetramethylammonium hydroxide (TMAH; Sigma Aldrich, USA). Several experiments using a certified reference material (Hay Powder, BCR 129; European Commission Joint Research Centre) with known iodine concentration (0.167 ± 0.024μg/g) were undertaken to develop the methodology in order to achieve optimal recovery of iodine based on the methods described by (Tagami et al., 2006). From these experiments, the optimal procedure was used: samples were shaken overnight in 10mL of 1.25% mass per volume (m/v) TMAH and heated at 60°C for 7h the next day. TMAH was identified as an appropriate method for extracting iodine from 500mg of the reference material.

Following extraction, 10 mL of Type I water and 0.2mL of internal standard (5 ppm Cs, Tb) were added to each the vial. Vials were then centrifuged at 3000 rpm for 10 minutes before 10mL of the supernatant was transferred into new vials to quantify by TqICP-MS. Recovery of iodine from BCR 129 was 100 ± 8% of the certified values.

### 2.8 Iodine analysis in extracted solutions

Detected iodine counts via TqICP-MS were converted to concentrations (µg/kg) based on the digestion factors (i.e., measured mass of sample used per mass of extractant solution). Standard solutions of iodine and blank solutions (0.625% m/v TMAH) were used to create the calibration curve. Several BCR 129 samples were also analyzed to ensure the accuracy of results. All calibration and BCR 129 solutions were prepared in the same way as grain samples, with equivalent additions of internal standards to correct for matrix effects and instrument drift. Triplicate grain iodine analyses suggested good reproducibility of iodine data with 2.81% the average relative standard deviations recorded. To assess the precision of results, some duplicate and quadruplicate grain samples were prepared and analyzed.

### 2.9 Statistical analysis

One-way analysis of variance was employed to determine statistically significant differences between different foliar adjuvant treatments on all dependent variables. Any significant differences were further analyzed using Tukey’s HSD *post-hoc* test. All tests had a significance level of p = 0.05. All analyses were performed in RStudio Version 2022.07.1 + 554 (RStudio Team, 2022).

## 3 Results

**Figure 1:**
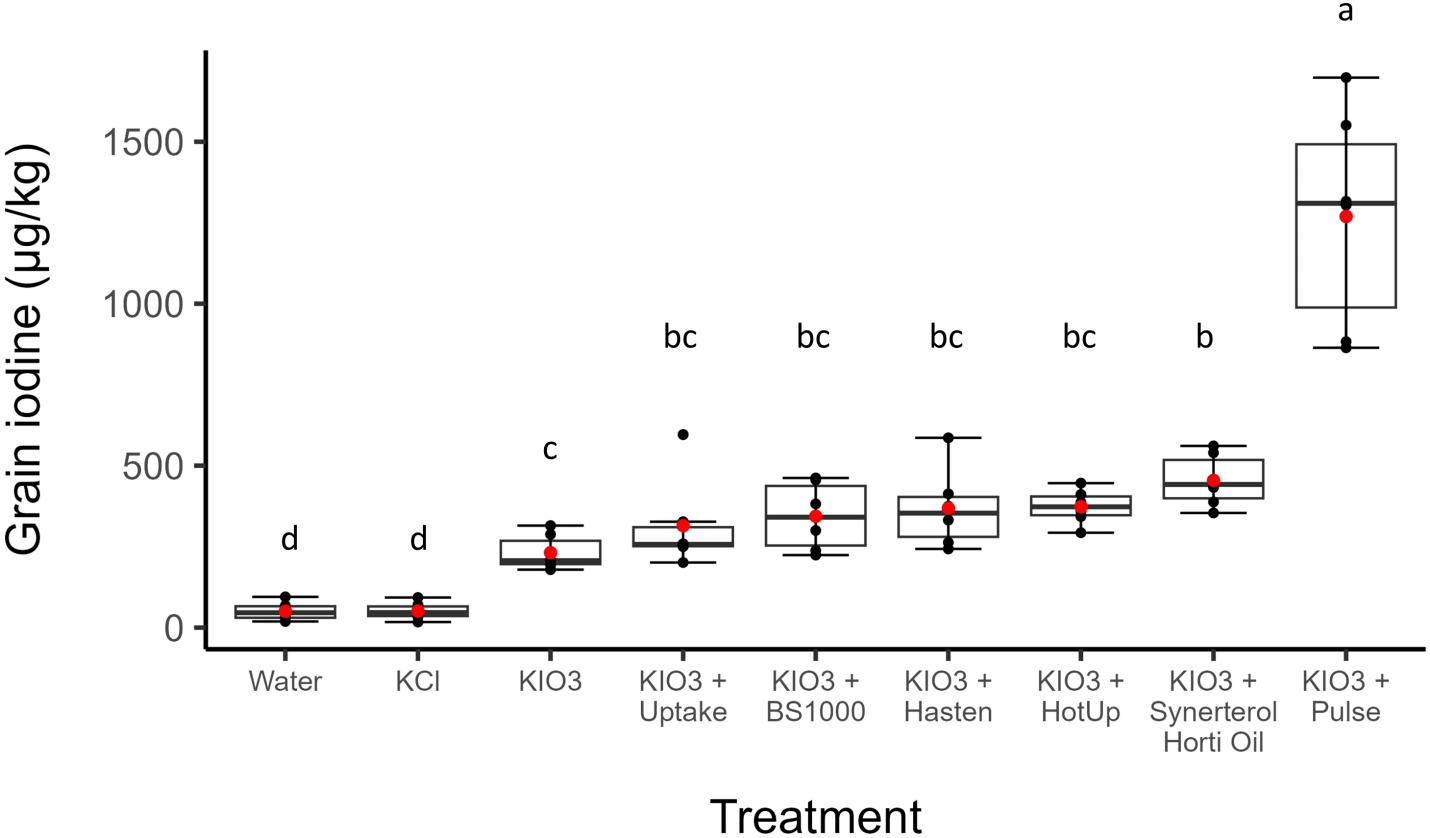
Iodine concentrations (µg/kg) of wheat grain across nine foliar spray treatments (n = 6). Horizontal lines indicate the median, boxes indicate the interquartile range, whiskers extend to the smallest and largest values within 1.5 times the interquartile range from the 25^th^ and 75^th^ percentiles, respectively, red dots indicate the mean. Differences in mean iodine concentrations are not significant, according to Tukey’s HSD test (p = 0.05), in treatment groups with the same letter.

Foliar application of 0.05% w/v KIO_3_ at heading and early milk growth stages of wheat plants increased the concentration of grain iodine in wheat (all p<0.05) but some spray adjuvants further improve the effectiveness of biofortification. All wheat treated with KIO_3_ produced grain with substantially higher iodine concentrations than wheat sprayed with water or KCl (both 51µg/kg, **Figure 1**). The application of KIO_3_ alone increased grain iodine from 51 to 231µg/kg. The addition of Synerterol^®^ Horti Oil (p < 0.01) and Pulse^®^ (p < 0.001) to the spray solution resulted in significantly higher iodine concentrations compared to the KIO_3_ control, with approximately 2-fold (231 to 455μg/kg) and 5-fold (231 to 1269μg/kg) increases in grain iodine, respectively. No other adjuvant had a significant effect on grain iodine compared to the KIO_3_ control treatment. Whilst Synerterol^®^ Horti Oil significantly increased grain iodine (455μg/kg) when compared to the KIO_3_ control (231μg/kg), the effect was not significantly different than the effect of Uptake^®^ (315μg/kg), BS1000 (344μg/kg), Hasten^®^ (369μg/kg), and Hot-Up^®^ (373μg/kg).

**Figure 2:**
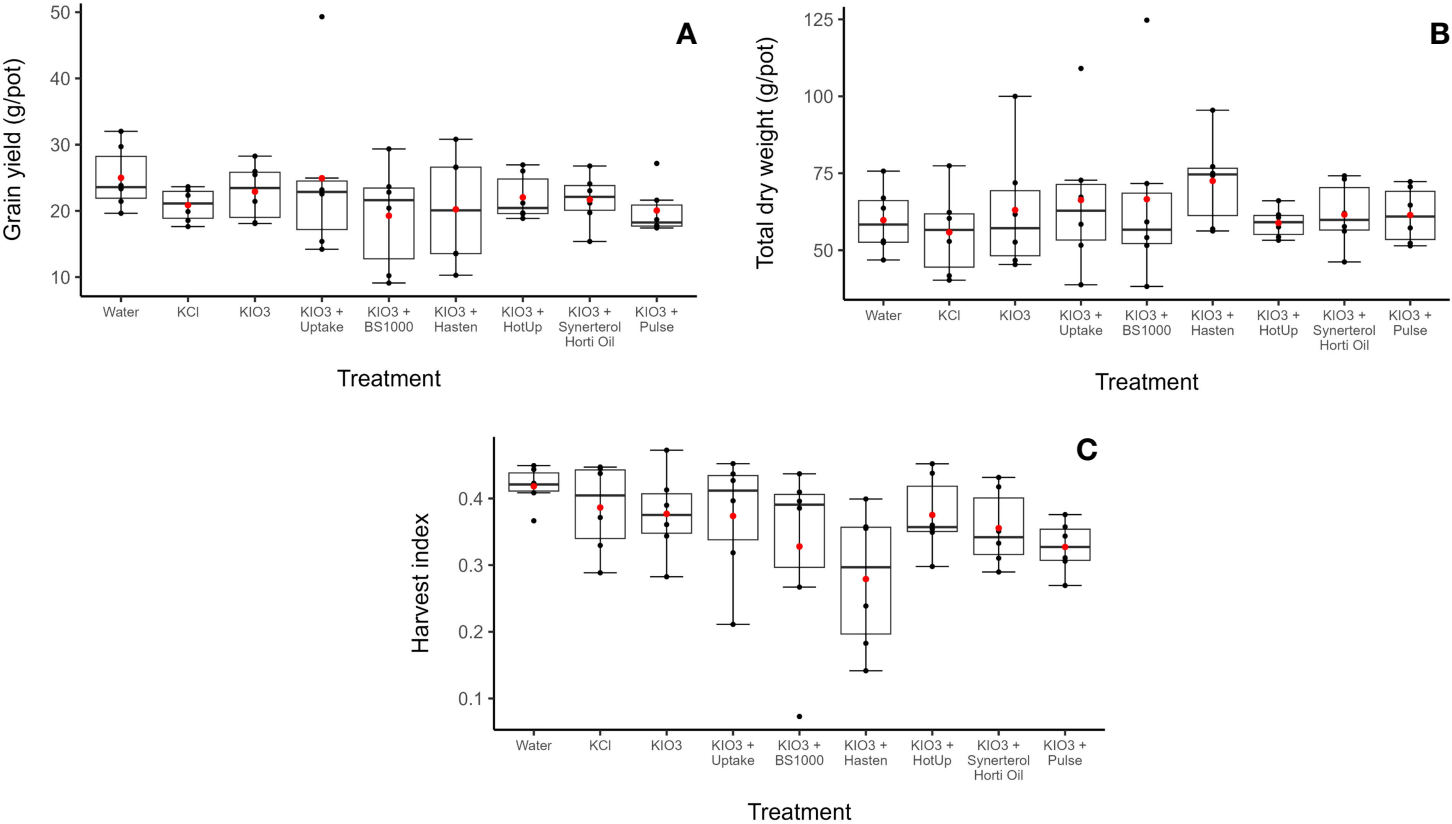
Wheat grain yield (g/pot) **(A)**, total dry weight (g/pot) of wheat **(B)** and wheat harvest index **(C)** across nine foliar spray treatment groups. Horizontal lines indicate the median, boxes indicate the interquartile range, whiskers extend to the smallest and largest values within 1.5 times the interquartile range from the 25th and 75th percentiles, respectively, red dots indicate the mean.

The foliar treatments and adjuvant treatments had no significant effects on grain yield (p > 0.05) (**Figure 2**). Other measurements of plant performance, including, total biomass production, and harvest index, showed no significant differences (p > 0.05) due to foliar spray treatment (**Figure 2**).

**Figure 3:**
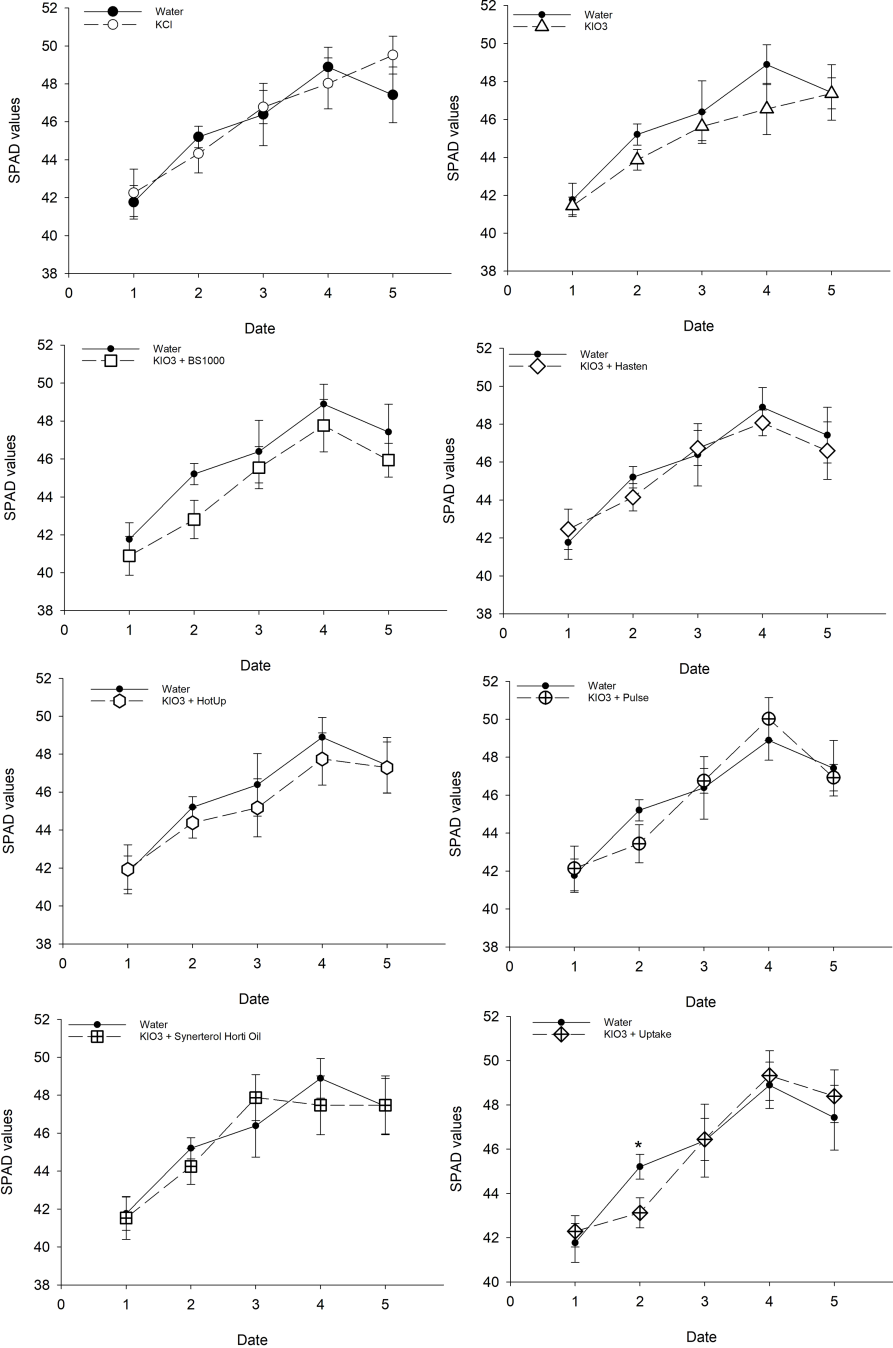
Means of three SPAD measurements (indicating the relative concentration of leaf chlorophyll) of six wheat leaves from each pot compared with the readings for the Water control, ± se. Each of the nine foliar spray treatments had six replicate pots. Measurements were recorded at 5 dates (1= prior to the first treatment application, 2 = one week post first application, 3 = prior to second application, 4 = approximately midway between first and second application, 5 = one week post second application).

Across the five timepoints in which measurements were conducted, there were no significant differences in SPAD values between iodine spray treatments and the control (Water) (**Figure 3**). A single exception was observed one week after the first application between KIO_3_ + Uptake (43.1) and Water (45.2), however, in the four other sampling times, no difference was observed between plants sprayed with KIO_3_ + Uptake and the Water control.

## 4 Discussion

Our results suggest for the first time that selected spray adjuvants further improve wheat’s loading of foliar-applied iodine into grain. The iodine concentration in grain from the KIO_3_ only treatment (231 ± 14.7μg/kg) was similar to the 273μg/kg obtained in a previous study with two applications of 0.05% w/v KIO_3_ (Cakmak et al., 2020). Cakmak et al. (2020) measured iodine concentrations of 71μg/kg in white bread and 211μg/kg in wholegrain bread prepared from the agronomically biofortified grain, suggesting a link between grain and bread concentrations, and that these concentrations were appropriate for correcting and preventing iodine deficiency in people with moderate to high levels of bread intake. Hence, with Pulse^®^ and Synerterol^®^ Horti Oil adjuvants there is potential to reduce the quantities of iodine needed to achieve the required iodine concentration in grain for successful biofortification. However, further study is needed on how grain iodine concentrations translate to wheat products such as bread.

Of all adjuvants, only Pulse^®^ and Synerterol^®^ Horti Oil significantly increased grain iodine compared to the KIO_3_ control. All adjuvants were applied at rates according to the label’s directions of use relevant to the situation that best matched the experimental conditions, resulting in varied application rates of active ingredients. None of the adjuvants tested are currently registered for grain biofortification purposes. However, in accord with the results presented, Pulse^®^ and Synerterol^®^ Horti Oil were the only products tested that are recommended for improving the performance of foliar fertilizers in addition to herbicides, fungicides, and insecticides (Nufarm, 2023a; Organic Crop Protectants, 2023).

The large increase in grain iodine with the application of Pulse^®^ may be attributed to the unique properties of organosilicons that improve the solid-liquid interactions at leaf surfaces. The primary advantage of organosilicons over other surfactants is the ability to promote stomatal infiltration and mass flow of foliar-applied solutions (Neumann and Prinz, 1974). In contrast, conventional surfactants act mostly by increasing cuticular penetration as they lower surface tension to a lesser extent (Stevens et al., 1992). Whilst all surfactants lower the surface tension of spray droplets and improve their spread across leaf surfaces, organosilicons reduce surface tension to a greater extent. Stomatal infiltration only occurs when the surface tension of a solutions is below the critical surface tension (Zisman, 1964) and the contact angle is smaller than the angle of pore walls (Schönherr et al., 2005), which organosilicons allow (Field and Bishop, 1988; Stevens, 1993; Zabkiewicz, 2000; Schönherr et al., 2005). Organosilicons have also been shown to improve the translocation of agrochemicals within plants due to stomatal infiltration allowing solutions to be deposited closer to vascular tissues where loading can occur (Stevens and Zabkiewicz, 1990; Stevens et al., 1992). The mechanisms of action for organosilicon need critical review as the only comprehensive review currently available (Knoche, 1994) is now nearly thirty years old.

By contrast with the present study, some reports suggest that organosilicons are not consistently more effective than conventional surfactants due to increased spreading of individual droplets leading to coalesce of droplets and subsequent run-off of spray solutions (Weinbaum and Neumann, 1977; Stevens, 1993) or rapid drying of spray droplets (Zabkiewicz et al., 1988; Field et al., 1992). Given the high iodine concentrations observed in grain, such loss is not expected to occur at organosilicon concentrations of up to 2 mg/L active ingredient. This current research indicates organosilicons do have an advantage over conventional surfactants and surfactant blends in the context of biofortifying wheat with iodine. They may also have a promising role in biofortification of other micronutrients in grain such as zinc, selenium and iron.

Organosilicons such as Pulse^®^ have been associated with increased phytotoxicity in various crops (Neumann and Prinz, 1974; Neumann and Prinz, 1975; Fernández et al., 2008). Anecdotally, organosilicons are suspected to destroy leaf cuticles. However, there is little published work that identifies the exact mode of action of phytotoxicity. Phytotoxicity may be caused by increased penetration of the active ingredient rather than direct effects of the adjuvant, as suggested by Orbović et al. (2007) who concluded mixing organosilicons with copper-based fungicides should be avoided in citrus crops due to toxic levels of copper ions penetrating through leaf and fruit cuticles. Yet other studies suggest that organosilicons are physiologically benign and less phytotoxic than other surfactants (Stevens and Zabkiewicz, 1990). The increased efficiency of organosilicons can also reduce the phytotoxic effects of agrochemicals by increasing the efficacy of lower application rates (Knoche, 1994). This is relevant to iodine, as high doses of iodine have been shown to adversely affect plant growth (Hong et al., 2008; Caffagni et al., 2012; Kato et al., 2013; Cakmak et al., 2017; Incrocci et al., 2019). Wheat’s iodine toxicity threshold is not well established, but Cakmak et al. (2017) observed leaf chlorosis and necrosis, reduced shoot dry matter production, and reduced grain yield in wheat treated with two foliar applications of iodine at concentrations above 2.3 mM, which corresponded to grain iodine concentrations in excess of 400 μg/kg. In the present trial, we did not observe any evidence of plant toxicity effects of increased iodine levels in terms of yield (**Figure 2**) or SPAD values (**Figure 3**).

The significant increase in grain iodine with the application of Synerterol^®^ Horti Oil is still unexplained as there are limited studies on the mode of action of oil-based adjuvants. Oils may improve foliar uptake of agrochemicals by increasing the retention time of sprays on leaf surfaces (Pacanoski, 2015). Botanical oil concentrates are the only active ingredient listed on the Synerterol^®^ Horti Oil label. Whilst the Synerterol^®^ Horti Oil was the only adjuvant tested that did not explicitly contain any non-ionic surfactant as an active ingredient (**Table 1**), botanical oil concentrates are defined by containing up to 20% non-ionic surfactant. Regardless, Synerterol^®^ Horti Oil is primarily comprised of canola oil, a renewable and potentially more sustainable resource than petroleum oils and synthetic surfactants. Increased use of products such as Synerterol^®^ Horti Oil should be considered as they are sustainably sourced, non-hazardous to humans, and often certified organic.

A further potential advantage common to all adjuvants is the reduced solute concentration on leaf surfaces leading to better penetration of IO_3_- across the cuticular membrane by lowering the relative humidity threshold. Below this threshold, foliar-applied solutions remain in the liquid state and form continuous connections from the outer to inner leaf cuticle to facilitate the diffusion of solutes. This function of formulation additives is described by Fernández et al. (2017). Alterations to spray solutions with the use of adjuvants such as Pulse^®^ and Synerterol^®^ Horti Oil is especially important for the effective penetration, and subsequent remobilisation, of iodine from water-soluble compounds such as KIO_3_ in wheat due to the hydrophobic, water-repellent character of wheat leaf surfaces (Fernández et al., 2014). Improved understanding of physical structure, chemical composition, and function of leaf surfaces would help optimise foliar uptake of iodine. Previously, radiotracing has studied the efficiency of foliar zinc fertilisers (Doolette et al., 2020) and future research involving the use of radioactively labelled iodine could provide insight into the mechanisms of short-distance mobility in the leaf and long-distance translocation from leaves to grains when iodine is applied to wheat in combination with different adjuvants.

High concentrations of iodine are toxic to living organisms, including both plants and humans. The grain iodine concentration achieved with Pulse^®^ (1269 ± 340μg/kg) is considerably above the 200 μg/kg required to adequately enrich food products made from biofortified wheat (Cakmak et al., 2017; Zou et al., 2019; Cakmak et al., 2020). Such concentrations may be unsafe for consumers. However, it suggests that successful agronomic biofortification of wheat can be achieved using less IO_3_ when applied with Pulse^®^, either by lowering the rates of KIO_3_ in solution or applying the 0.05% w/v KIO_3_ once only. Reducing the number of applications would have the added benefit of minimizing costs of application while improving the practical feasibility of implementing biofortification in grain production. However, further study is needed to identify if higher grain iodine concentrations will translate to higher end-user products, as the additional iodine might be allocated to parts of the grain that are removed during milling or baking processes. Defining biofortification protocols is important to ensure the final concentration of iodine in food remains within the narrow range of dietary requirements in relation to the volume consumed.

Whilst non-ionic surfactants have been suggested to improve the efficiency of foliar biofortification in wheat, the results of this experiment show that some other adjuvants classified in different categories based on their chemical composition were effective at increasing grain iodine concentrations. Identification of Pulse^®^ (an organosilicon) and Synerterol^®^ Horti Oil (a botanical oil concentrate) as useful adjuvants for enhancing foliar uptake and accumulation of iodine in wheat grain has important implications regarding the improvement of agronomic practices intended for grain biofortification. With the use of such adjuvants, the application of inorganic iodine can be optimised, translating to economically viable and environmentally sustainable agronomic biofortification.

Biofortifying Australian wheat with iodine will most likely increase the population’s iodine intake, reduce the burden of iodine deficiency and improve human health. As large quantities of wheat are included in the daily diet of many around the world, it could considerably reduce the prevalence of iodine deficiency without any need to actively change consumer behaviour. Improving the nutritional quality of wheat will support the viability of Australia’s export grain industry; grains with low concentrations of micronutrients may be at risk of international penalties or being unacceptable for export in the future. Furthermore, cost-effective agronomic biofortification of wheat with iodine may enhance profitability of grain production if increased micronutrient concentrations begin to attract premium prices.

## 5 Conclusion

The success of organosilicon and botanical oil concentrate adjuvants in increasing the efficacy of iodine foliar sprays is a promising finding for future biofortification efforts, as it could help alleviate dietary iodine deficiencies in populations consuming Australian wheat. To validate the findings of this experiment, the effect of adjuvants should be further tested to determine the appropriate application rate or the ideal number and timing of application of foliar iodine under field conditions.

## Data availability statement

The raw data supporting the conclusions of this article will be made available by the authors, without undue reservation.

## Author contributions

EM designed the experiment, carried out the data analysis and prepared the draft manuscript. MW, HW, TC, JS-P, RB and BP interpreted the result and edited the manuscript. All authors contributed to the article and approved the submitted version.
